# Genomic Insights into the Probiotic Functionality and Safety of *Lactiplantibacillus pentosus* Strain TBRC 20328 for Future Food Innovation

**DOI:** 10.3390/foods14172973

**Published:** 2025-08-26

**Authors:** Tayvich Vorapreeda, Tanapawarin Rampai, Warinthon Chamkhuy, Rujirek Nopgasorn, Siwaporn Wannawilai, Kobkul Laoteng

**Affiliations:** 1 Biosciences and Systems Biology Research Team, Biochemical Engineering and Systems Biology Research Group, National Center for Genetic Engineering and Biotechnology (BIOTEC), National Science and Technology Development Agency (NSTDA), Bangkok 10150, Thailand; tayvich.vor@biotec.or.th; 2 Industrial Bioprocess Technology Research Team, Functional Ingredients and Food Innovation Research Group, National Center for Genetic Engineering and Biotechnology (BIOTEC), National Science and Technology Development Agency (NSTDA), Pathum Thani 12120, Thailand; tanapawarin.ram@biotec.or.th (T.R.); warinthon.cha@biotec.or.th (W.C.); rujirek.nop@biotec.or.th (R.N.); siwaporn.wan@biotec.or.th (S.W.)

**Keywords:** *Lactiplantibacillus pentosus*, comparative genome analysis, exopolysaccharides, polyketide sythase, bacteriocin, short-chain fatty acid, whole genome sequencing

## Abstract

*Lactiplantibacillus* species have been historically used for food applications. Although several species are regarded as safe according to their regulatory status, the safety issues and functional roles of these lactic acid bacteria have been given attention. A selected *Lactiplantibacillus* strain TBRC 20328, with probiotic properties isolated from fermented Isan-style pork sausage (Mam), was evaluated for its safety through whole-genome sequencing and analysis using integrative bioinformatics tools. The metabolic genes were assessed through comparative genome analysis among *Lactiplantibacillus* species. The genome of the strain TBRC 20328 consisted of one circular chromosome (3.49 Mb) and five plasmids (totaling 0.25 Mb), encoding 3056 and 284 protein-coding genes, respectively. It exhibited an average nucleotide identity (ANI) with other *Lactiplantibacillus pentosus* strains of over 95%. Whole-genome analysis confirmed the absence of virulence and antimicrobial resistance genes, supporting its safety for food applications. Functional annotation revealed clusters for bacteriocins (plantaricin EF and pediocin) and polyketides, indicating potential roles in biopreservation and host interactions. Genes involved in the biosynthesis of some short-chain fatty acids and exopolysaccharides were also identified. Comparative genomic analysis across 33 other *Lactiplantibacillus* strains identified 2380 orthogroups, with 94 unique to the *Lp. pentosus* group. These included gene clusters involved in malonate decarboxylation, leucine biosynthesis, and 5-oxoprolinase activity. Such distinct genomic features emphasize the sustainable biotechnological potential and safety of *Lp. pentosus* TBRC 23028. Together, the findings highlight its promise as a safe and functional probiotic candidate with broad applications in functional food development and precision fermentation technologies.

## 1. Introduction

Probiotic bacteria are live microbes that, when carefully administered, contribute to host well-being. These microorganisms typically survive harsh conditions such as stomach acid and bile salts, adhere to the intestinal lining, and influence immune and barrier functions. Such characteristics made them invaluable in preventing gastrointestinal disorders, maintaining microbial balance, and supporting health beyond digestion. Among probiotic genera, *Lactobacillus* species, which are lactic acid bacteria (LAB), are widely known for their roles in food preservation and health promotion, which are achieved through traditional and precision fermentation technologies. The genus *Lactiplantibacillus*, such as *Lactiplantibacillus plantarum*, *Lactiplantibacillus paraplantarum*, and *Lactiplantibacillus pentosus*, is particularly notable for its broad applications in starter cultures, probiotics, and functional ingredients that offer significant health benefits [1,2]. These species are commonly isolated from a variety of fermented foods, such as olives, sourdough, and pickled vegetables, where they play essential biological functions [3,4]. Their metabolic activities enhance the complex flavors by producing organic acids and aroma compounds, and improve texture through structural and enzymatic modifications. Additionally, their growth enriches the nutritional value of foods by synthesizing bioactive compounds, including vitamins, exopolysaccharides, and antimicrobial peptides [5,6].

Among these species, *Lp. pentosus* is known for its exceptional ability to survive and thrive in harsh environmental conditions, such as high salinity, acidic pH level, and nutrient scarcity [7]. The cell resilience makes it especially valuable in industrial applications, particularly in the production process of fermented foods, where it ensures reliable performance under variable conditions [1,4]. It has been reported that certain *Lp. pentosus* strains possess significant potential for innovative biotechnological applications, including the development of functional foods, waste valorization, and the production of natural preservatives [8]. They can produce a variety of bioactive compounds, including antimicrobial peptides and exopolysaccharides (EPSs), that contribute to food preservation, enhance texture, and provide functional benefits in food applications [9]. These biomolecules contribute collectively to the functional dynamics of microbial consortia in waste fermentation systems. EPS supports the formation of structured biofilms that promote the retention and proximity of degradative enzymes and microbial populations, while AMPs selectively suppress competing or harmful microbes, thereby improving microbial efficiency, ecological balance, and system resilience during organic waste processing [10,11]. Moreover, the ability of *Lp. pentosus* to degrade certain toxins and inhibit the growth of harmful pathogens further strengthens its role in biopreservation [7]. Additionally, *Lp. pentosus* is renowned for its probiotic properties, which support gut health and boost the immune system. With its multifunctional properties and industrial significance, *Lp. pentosus* remains a central focus of fundamental and applied research in food biotechnology and related fields. However, *Lp. pentosus* strains exhibit diverse traits, with each strain demonstrating distinct functional capabilities and environmental adaptability, largely influenced by their genetic backgrounds, which can influence phenotypic traits, such as probiotic potency, metabolic pathways, antimicrobial activity, and stress tolerance capacity. With the advancement of next-generation sequencing technologies, exploring the genetic diversity of *Lactiplantibacillus* strains provides comprehensive insights into strain-specific characteristics by identifying genetic barcodes associated with desired traits and revealing novel metabolic pathways. Not only used for investigating evolutionary relationships and functional diversity among closely related species, comparative omics studies also facilitate the optimization of bioprocesses and uncover the full potential of *Lactiplantibacillus* strains, enabling the development of tailored solutions across various applications.

Previously, we identified an *Lp. pentosus* isolate (TBRC 20328) from fermented Isan-style pork sausage (Mam). The experimental investigations of fundamental probiotic properties were conducted, demonstrating the strain’s promising potential, including acid and bile tolerance, and adhesion to Caco-2 intestinal cells. The DPPH assay [12] and ELISA targeting tumor necrosis factor-alpha (TNF-α) secretion in THP-1 macrophages [13] showed that the strain possesses antioxidant and anti-inflammatory activities, respectively. Using the agar well diffusion method [14], the TBRC 20328 strain also exhibited antimicrobial activity against both Gram-positive bacteria (*Streptococcus gordonii*, *S. pyogenes*, *S. mutans*, *Staphylococcus aureus*, *Listeria monocytogenes*, and *Propionibacterium acnes*) and Gram-negative bacteria (*Salmonella Typhimurium* and *Helicobacter pylori*). These multifunctional properties highlight the strain’s technological relevance, supporting its potential application in the development of functional foods, natural preservatives, and probiotic formulations. Nevertheless, further investigation is needed to determine its safety and to explore its additional functional properties. In this study, the genome sequencing and characterization of the probiotic *Lp. pentosus* TBRC 20328 was conducted. The safety profile of the selected strain, TBRC 20328, was assessed using computational analysis, ensuring its suitability for industrial applications. The metabolic genes and pathways associated with growth behaviors, as well as additional probiotic functions, including bacteriocin, short-chain fatty acid (SCFA), and EPS biosynthesis, were systematically analyzed through a comparative genomic study. The findings offer valuable insights into the systematic development of this strain and its production process, driving the innovative creation of functional food ingredients, healthy foods, and other bioproducts. This study suggests beneficial possibilities for utilizing precision fermentation and synthetic biology approaches to enhance the efficiency and sustainability of targeted production.

## 2. Materials and Methods

### 2.1. Bacterial Strain and Cultivation Condition

*Lp. pentosus* strain TBRC 20328, isolated from the fermented Isan-style pork sausage (Mam) and deposited in the Thailand Bioresource Research Center (TBRC), was used in this work. It was cultivated in MRS broth at 37 °C overnight. The cells were harvested by centrifugation at 8000–10,000 rpm for 15–20 min for further extractions of plasmid and genomic DNA.

### 2.2. Plasmid and Genomic DNA Extractions

The genomic DNA of *Lp. pentosus* was extracted using a Wizard Genomic DNA Purification kit (Promega Corporation, Madison, MI, USA) following the manual’s instructions. The DNA concentration and quality were measured using a NanoDrop ND-1000 spectrophotometer (Thermo Fisher Scientific, Waltham, MA, USA).

The plasmid DNA of *Lp. pentosus* was extracted using a ZymoPURE Plasmid Miniprep kit (Zymo Research Corporation, Tustin, CA, USA) following the manual’s instructions. Then, the number and size of plasmids were analyzed by gel electrophoresis using a 0.5% agarose gel in TBE buffer.

### 2.3. Whole-Genome Sequencing and Genome Assembly

The genome of *Lactiplantibacillus* strain TBRC 20328 was sequenced using the PacBio RSII SMRT cell platform at McGill University and Génome Québec Innovation Centre, Canada. The sequencing reads were assembled de novo using the Celera Assembler within the hierarchical genome assembly process (HGAP) workflow [15].

### 2.4. Gene Prediction and Functional Annotation

Gene prediction and computational annotation of protein-coding genes were performed using Prokka (v.1.14.6) [16] with default parameter settings. To achieve functional annotation, the protein sequences generated by Prokka were utilized through precomputed orthology assignments, employing the EggNOG-mapper tool (v.2.1.12) [17]. Subsequently, these protein sequences were searched against the EggNOG database (version 5) [18] using the DIAMOND protein aligner [19]. Gene prediction and computational annotation of protein-coding genes were performed using Prokka [16] with default parameter settings. To achieve functional annotation, the protein sequences generated by Prokka were utilized through precomputed orthology assignments, employing the EggNOG-mapper tool (v.2.1.12) [17]. Subsequently, these protein sequences were searched against the EggNOG database (version 5) [18] using the DIAMOND protein aligner [19].

### 2.5. Species Identification

The initial identification of the strain’s species was implemented by analyzing the 16S rRNA gene sequences. A set of 16S rRNA gene copies was screened and extracted from the genomic data available in the National Center for Biotechnology Information (NCBI) database using the Basic Local Alignment Search Tool (BLAST v.2.16.0). After confirming all sequences of *Lactobacillaceae*, these sequences were utilized to construct a phylogenetic tree using the Molecular Evolutionary Genetic Analysis (MEGA 11 v.11.0.11) software [20].

To further validate the species classification, average nucleotide identity (ANI) was computed against the type strains of the designated species using the OrthoANI method [21]. The species categorization was confirmed based on a threshold ANI value of ≥95–96%, adhering to the criteria established in the previous report [22].

### 2.6. Determination of Antimicrobial Resistance (AMR), Virulence Factors (VFs), Antibiotic Resistance Genes (ARG), and Undesirable Genes

In silico analyses of AMR, VFs, and ARG were performed. The protein-coding genes of the *Lp. pentosus* TBRC 20328 genome were searched for antimicrobial resistance and virulence factors using seven specialized databases. These included the Comprehensive Antibiotic Resistance Database (CARD) [23], Antibiotic Resistance Gene-ANNOTation (ARG-annot) [24], Virulence Factor Database (VFDB) [25], ResFinder [26], MEGARes [27], the NCBI-AMRFinder [28], and PlasmidFinder [29]. This comprehensive screening was performed using ABRicate (version 1.0.1) [30] to identify annotated replicons and genetic elements related to AMR, virulence factors, and undesirable genes.

### 2.7. Biosynthesis Gene Cluster Analysis

To identify gene clusters responsible for the biosynthesis of secondary metabolites across various chemical classes in the *Laciplantibacillus* genome, we performed in silico analysis using antiSMASH 7.1.0 (Antibiotics and Secondary Metabolite Analysis Shell) [31] with default parameters. PRISM 4 (Prediction Informatics for Secondary Metabolomes) [32], available at http://prism.adapsyn.com/ (accessed on 24 March 2024), was used with default settings to further identify biosynthetic gene clusters. Bacteriocin operons within the genome were also examined using the BActeriocin GEnome mining tooL (BAGEL4) [33].

The genome sequences were prepared in FASTA format and analyzed using OrthoFinder (version 2.5.5) [34] for predicting orthologous gene groups across multiple genomes. OrthoFinder, utilizing the DIAMOND alignment algorithm, was employed to conduct high-speed and accurate sequence comparisons, clustering similar proteins into orthogroups that represent putative gene families, thereby elucidating evolutionary and functional relationships among genes.

The OrthoFinder analysis was conducted using default parameters, enabling the identification and classification of orthologous genes in a dataset that included the genome of *Lactiplantibacillus* strain TBRC 20328 along with 32 additional genomes from closely related *Lactiplantibacillus* species. This dataset comprised 12 *Lp. pentosus*, 13 *Lp. plantarum*, and 7 *Lp. paraplantarum* genomes, all sourced from the NCBI database. By including such diverse genomes, this analysis elaborated a comprehensive framework for exploring genetic conservation and divergence within the genus, providing valuable insights into the evolutionary dynamics and functional diversity of *Lactiplantibacillus* species.

## 3. Results and Discussions

### 3.1. Genome Features and Species Identification of Lactiplantibacillus TBRC 20328

**Figure 1:**
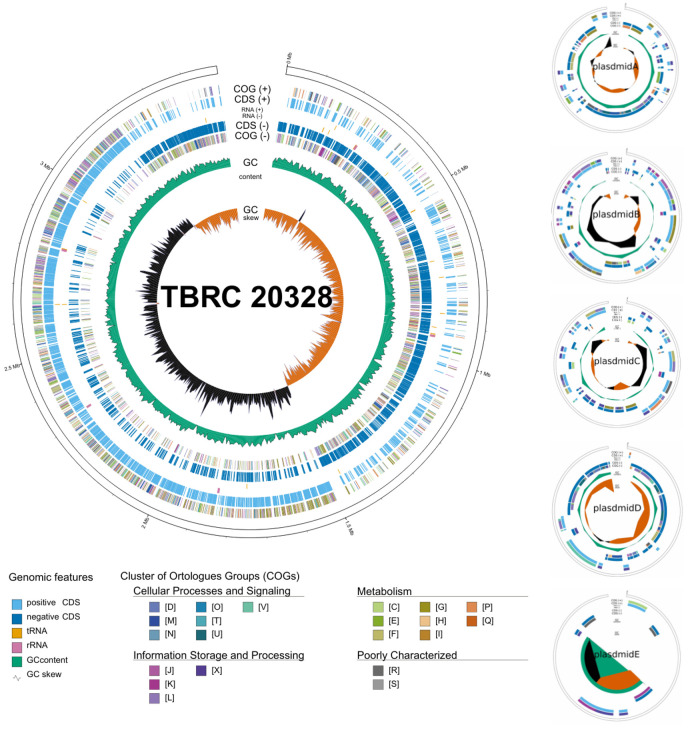
A circular map of the *Lactiplantibacillus* TBRC 20328 genome was generated using GenoVi v04.3 [40]. The primary map (left-hand side) displays the chromosome and five plasmids (A–E) illustrated on the right-hand side. The genomic features and Clusters of Orthologous Groups (COGs) were identified through PROKKA annotation and are visualized on the genome (from outer to inner): circles 1 and 2 are color-coded based on the COG classification and represent coding sequences (CDSs) located on the forward strand; circles 3 and 4 are color-coded based on the RNA classification, representing features on the forward and reverse strands, respectively; circles 5 and 6 are color-coded based on the CDS and COG classification, representing features on the reverse strand; circles 7 and 8 display GC content and GC skew, respectively.

The genome of *Lactiplantibacillus* TBRC 20328 consisted of 3,491,384 base pairs (bp) and a GC content of 46.60%. It contained five plasmids labeled A to E, ranging in size from 70,426 bp to 6885 bp, as shown in Table 1 and Figure 1. Genome annotation using Prokka [16] identified 3136 genes, of which 3056 protein-coding sequences were annotated. The computational analysis revealed 63 transfer RNA (tRNA) genes, 16 ribosomal RNA (rRNA) genes, and one transfer-messenger RNA (tmRNA). The rRNA genes contained five copies of each of the 16S and 23S rRNA genes and six copies of the 5S rRNA genes. Assembly completeness was assessed using Benchmarking Universal Single-Copy Orthologs (BUSCO) v.5 [35] through the gVolante server [36] and CheckM [37]. Using a reference gene set from *Lactobacillales*, BUSCO analysis showed 99.8% completeness. CheckM also estimated completeness at 99.38% based on *Lactobacillales* marker sets, with a contamination level of 2.01%, as detailed in Supplementary Data Table S1.

**Figure 2:**
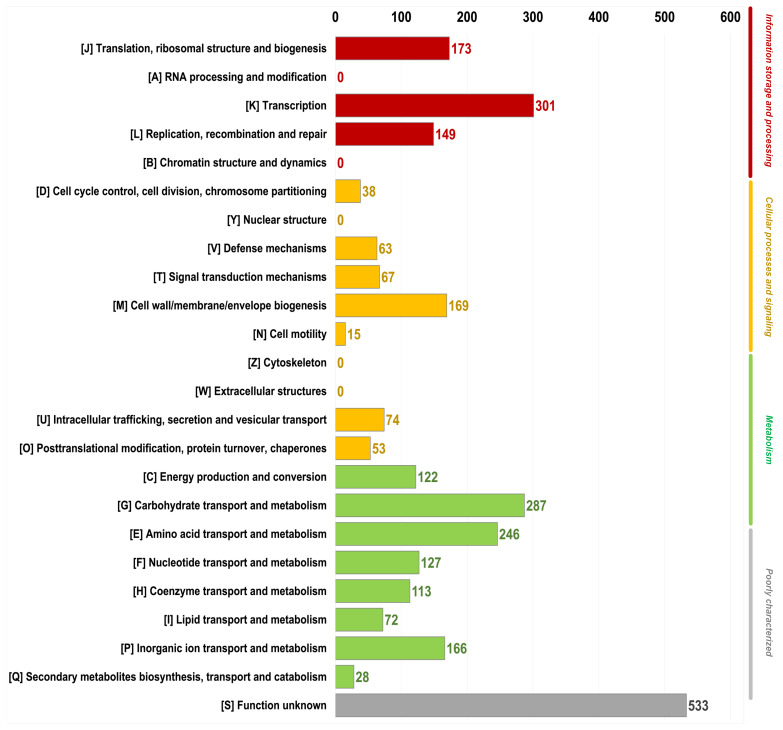
Functional Category of putative protein-coding genes in the *Lactiplantibacillus* TBRC 20328 genome. Numbers indicate the number of genes in each category.

Furthermore, the annotated genes were compared against the Clusters of Orthologous Groups (COG) [38] and Kyoto Encyclopedia of Genes and Genomes (KEGG) databases [39] to gain insight into their biological functions. The functional categorization of the putative protein-coding genes of *Lactiplantibacillus* TBRC 20328 is illustrated in Figure 2. The genes of the *Lactiplantibacillus* TBRC 20328 chromosome were categorized into 18 functional groups based on their assignment to Clusters of Orthologous Groups (COG) families and a “function unknown” category [S], which consisted of 533 genes. Most of the genes in *Lactiplantibacillus* TBRC 20328 were associated with essential cellular functions. Notably, 301 genes were classified under the transcription category [K], suggesting a significant reliance on gene regulation and expression in various cellular processes. A substantial number of genes, 287 in total, were associated with carbohydrate transport and metabolism [G], while 246 genes were involved in amino acid transport and metabolism [E]. Together, these categories [G and E] underscore the essential role of carbohydrate and amino acid pathways in cellular functions. Additionally, 173 genes were associated with translation, ribosomal structure, and biogenesis [J], emphasizing the importance of protein synthesis for growth and cellular function. A total of 169 genes related to the cell wall, membrane, and envelope biogenesis [M] were identified, suggesting that maintaining structural integrity is essential for *Lactiplantibacillus* TBRC 20328. Only 15 genes were functionally assigned to cell motility [N], indicating that this function is minimally represented in the genome.

Furthermore, the annotated genes were compared against the Clusters of Orthologous Groups (COG) [38] and Kyoto Encyclopedia of Genes and Genomes (KEGG) databases [39] to gain insight into their biological functions. The functional categorization of putative protein-coding genes of *Lactiplantibacillus* TBRC 20328 is illustrated in Figure 2. The genes of the *Lactiplantibacillus* TBRC 20328 chromosome were categorized into 18 functional groups based on their assignment to Clusters of Orthologous Groups (COG) families and a “function unknown” category [S], which consisted of 533 genes. Most of the genes in *Lactiplantibacillus* TBRC 20328 were associated with essential cellular functions. Notably, 301 genes were classified under the transcription category [K], suggesting a significant reliance on gene regulation and expression in various cellular processes. A substantial number of genes, 287 in total, were associated with carbohydrate transport and metabolism [G], while 246 genes were involved in amino acid transport and metabolism [E]. Together, these categories [G and E] underscore the essential role of carbohydrate and amino acid pathways in cellular functions. Additionally, 173 genes were associated with translation, ribosomal structure, and biogenesis [J], emphasizing the importance of protein synthesis for growth and cellular function. A total of 169 genes related to cell wall, membrane, and envelope biogenesis [M] were identified, suggesting that maintaining structural integrity is essential for *Lactiplantibacillus* TBRC 20328. Only 15 genes were functionally assigned to cell motility [N], indicating that this function is minimally represented in the genome.

**Figure 3:**
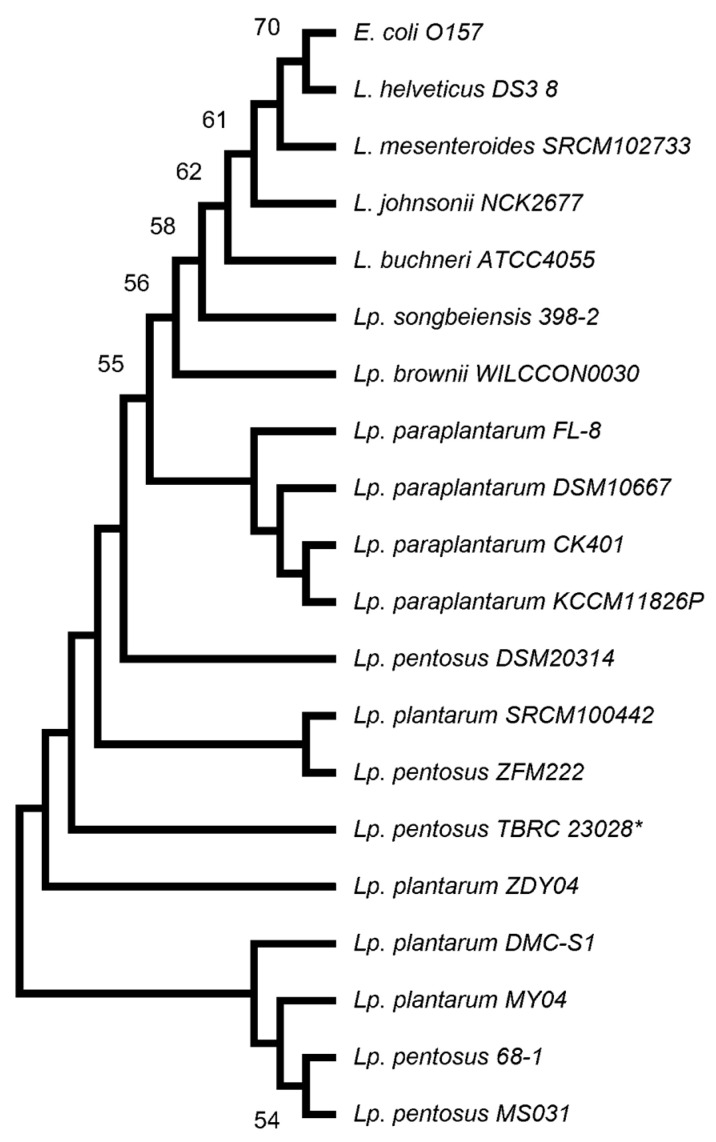
A phylogenetic tree was constructed using the neighbor-joining method in MEGA 11, based on the 16S rRNA gene sequence of 19 *Lactobacillaceae* strains. *Escherichia coli* is used as an outgroup. Bootstrap values, derived from 1000 resamplings, are indicated at branching points where values exceeded 50%. The scale bar represents 0.01 substitutions per nucleotide position. The strain used in this study, *Lp. pentosus* TBRC 20328, is marked with an asterisk (*).

**Figure 4:**
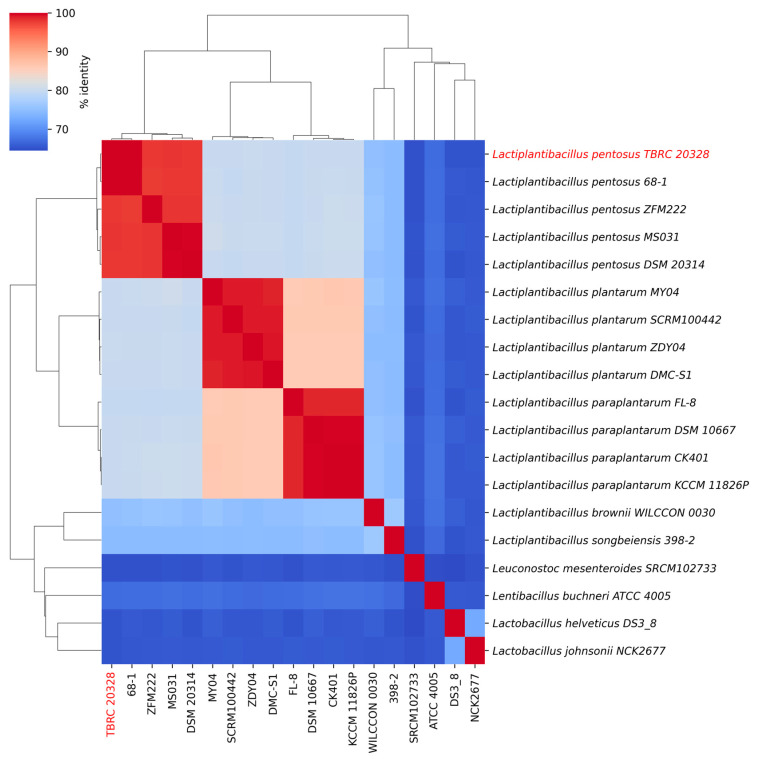
The hierarchical clustering and heatmap display average nucleotide identity (ANI) values across various strains of *Lp. pentosus*, *Lp. plantarum*, and *Lp. paraplantarum*, as well as other genera from the *Lactobacillaceae* family. The heatmap specifically illustrates the ANI values for strain TBRC 20328 in relation to closely associated *Lp. pentosus* strains.

The 16S rRNA gene sequences of *Lactiplantibacillus* TBRC 20328 exhibited 100% identity with those of other *Lactiplantibacillus* species, including *Lp. plantarum* SRCM100442, *Lp. plantarum* DMC-S1, *Lp. pentosus* ZFM222, and *Lp. pentosus* strain 68-1. All similarity values were observed to surpass the 98.7% cut-off threshold proposed for species-level identification [41]. To further clarify evolutionary relationships, a phylogenetic analysis was performed using the Molecular Evolutionary Genetics Analysis program (v. 11). The evolutionary tree constructed based on 16S rRNA sequences from members of the *Lactobacillaceae* family is illustrated in Figure 3. Using multiple sequence comparisons by ClustalW with the maximum composite likelihood method, the sequence alignment results indicate that the 16S rRNA gene analysis could not effectively distinguish between *Lp. plantarum* and *Lp. pentosus* within this bacterial group. To overcome the limitation and ensure accurate species assignment, whole-genome sequence data for the strain TBRC 20328 were intensively analyzed. The ANI value calculation revealed that the strain TBRC 20328 shared the highest ANI value of 99.89% with *Lp. pentosus* strain 68-1, which is shown in Figure 4. ANI values for other *Lp. pentosus* strains also exceeded the 95–96% species delineation threshold as proposed by Richter and Rosselló-Móra (2009) [22]. In contrast, ANI values of other *Lp. plantarum* and *Lp. paraplantarum* species were notably lower, falling below 81%. These findings, supported by genome-level data, strongly confirm that the strain TBRC 20328 belongs to the species *Lactiplantibacillus pentosus*. This whole-genome approach demonstrated superiority over 16S rRNA analysis for resolving closely related taxa within the *Lactobacillaceae* family.

### 3.2. Absence of Antimicrobial Resistance Genes (AMR), Virulence Factors (VFs), Antibiotic Resistance Genes (ARG), and Mobile Genetic Elements

Using ABRicate (version 1.0.1) [30] with default parameters, the *Lp. pentosus* TBRC 20328 genome was screened against databases for AMR, VFs, ARG, and undesirable genes. The analysis revealed no detectable AMR, VFs, ARG, or undesirable genetic elements. Notably, no such genes were identified within plasmids or near plasmid replicon regions. These results suggest that the strain TBRC 20328 poses minimal safety risks for food applications, as the absence of functional or transferable AMR genes supports regulatory compliance and reduces concerns about horizontal gene transfer or pathogenicity. By avoiding the pitfalls associated with antimicrobial resistance and pathogenicity, this strain holds promise for the development of sustainable and safe food solutions. Future studies should focus on the long-term evaluations of its functional properties across diverse food matrices, as well as its interactions with native gastrointestinal microbiota, to further substantiate its safety and efficacy.

Mobile genetic elements play a crucial role in organizing bacterial genomes by facilitating horizontal gene transfer, which enables functional adaptation and contributes to genomic stability. Careful consideration of the safety and functionality of bacterial probiotics and starter strains is essential, as these are critical factors in functional food applications. Thus, the MGE analysis is mainly required for seeking genes associated with antimicrobial resistance, metabolic traits, or niche-specific adaptation. To discover prophage elements in the genome of *Lp. pentosus* TBRC 20328, we employed the PHAge Search Tool Enhanced Release (PHASTER), available at https://phastest.ca (accessed on 19 August 2024), to thoroughly examine potential prophage regions. This tool provided insights into their length, location, GC content, and annotated genes [42]. Despite using both ‘lite’ and ‘deep’ annotation modes, the analysis found no evidence of prophage fragments within the TBRC 20328 genome, indicating an absence of integrated viral sequences. Additionally, the PlasmidFinder tool could identify two plasmid replicons, rep38_2_repA (LBPp1) and rep38_1_rep (pLBUC03), in the TBRC 20328 strain, as shown in Table 2. These replicons have been previously annotated in *Lp. plantarum* P-8 and *L. buchneri* NRRL B-30929, respectively. The results highlight the presence of functional plasmid elements and confirm the absence of integrated prophage sequences in strain TBRC 20328, underscoring its genomic stability and suitability for industrial applications. Plasmids can confer adaptive advantages, such as metabolic versatility and stress resilience, thereby increasing strain robustness under industrial processing conditions and supporting its application in fermentation-based processes [43]. Moreover, the absence of prophages reduces the risk of horizontal gene transfer to other microorganisms, whether in food products or within the human gut microbiota, further enhancing the safety profile of *Lp. pentosus* TBRC 20328 and increasing consumer confidence in its use.

### 3.3. Biogenic Amine Biosynthesis Genes

LAB plays a pivotal role in producing biogenic amines (BAs) with the metabolic activity of amino acid decarboxylation. While BAs contribute to the distinctive flavors and characteristics of fermented products, they can also pose health risks. For some individuals, consuming foods high in biogenic amines may trigger adverse reactions, such as headaches, heart palpitations, vomiting, and diarrhea [44,45]. The severity of these symptoms can vary widely among individuals, emphasizing the importance of understanding the effects of LAB and their metabolic byproducts on food safety and human health. Selecting probiotic bacteria that do not produce BA is crucial for fermentation. In this study, we evaluated the presence of essential genes involved in the biosynthetic pathways of BAs in the genome of *Lp. pentosus* TBRC 20328 through amino acid sequence similarity searches. The analysis revealed the absence of several key genes associated with BAs production [46] in the TBRC 20328 genome, including lysine decarboxylase (EC: 4.1.1.18), ornithine/lysine decarboxylase (EC: 4.1.1.116), arginine decarboxylase (EC: 4.1.1.19), agmatinase (EC: 3.5.3.11), spermidine synthase (EC: 2.5.1.16), spermine synthase (EC: 2.5.1.22), arginase (EC: 3.5.3.1), ornithine decarboxylase (EC: 4.1.1.17), histidine decarboxylase (EC: 4.1.1.22), tyrosine decarboxylase (EC: 4.1.1.25), and tryptophan decarboxylase (EC: 4.1.1.28). This finding suggests the favorable safety profile of *Lp. pentosus* TBRC 20328 for consumption due to the genomic absence of critical genes involved in biogenic amine synthesis, highlighting its suitability for food-related applications. This genetic feature reduces concerns about toxic metabolite accumulation, reinforcing its promise as a stable and safe microbial resource for fermentation-based innovations.

### 3.4. Bile Salt Deconjugations

The ability to hydrolyze bile salts is a key criterion for probiotic selection. However, selecting effective probiotic strains that can function optimally in the gastrointestinal tract remains a significant challenge. We identified two genes within the genome of *Lp. pentosus* TBRC 20328 that are homologous to the choloylglycine hydrolase family and linked explicitly to bile salt hydrolase (BSH; cholylglycine hydrolase; and EC 3.5.1.24). BSH is an enzyme produced by LAB, such as *Lactiplantibacillus* and *Bifidobacterium* strains, playing a crucial role in bile acid metabolism [47,48] by catalyzing the hydrolysis of bile salts conjugated with amino acids, including glycine and taurine, thereby disrupting the formation of cholesterol micelles necessary for intestinal cholesterol absorption. This function can reduce cholesterol uptake and improve lipid profiles [49]. Thus, bile salt hydrolase in the TBRC 20328 strain could enhance its tolerance to bile acids and facilitate bile salt hydrolysis, suggesting its potential for cholesterol management. Recent studies highlighted the potential of bile salt deconjugation by LAB as a therapeutic approach to lower serum cholesterol levels in hypercholesterolemic patients and to prevent hypercholesterolemia in individuals with normal cholesterol levels [50].

### 3.5. D-Lactic Acid Production

Using the KEGG database searching, the lactate racemase (Lar) and D-lactate dehydrogenase (LDHD) genes were identified in the genome of *Lp. pentosus* TBRC 20328. These enzymes catalyze the production of D-lactic acid, a key component of cell wall peptidoglycan in several Gram-positive bacteria, including the *Lactiplantibacillus* genus [51]. Since D-lactic acid production is an intrinsic property of these bacteria, precautions should be taken when consuming high amounts of D-lactic acid-producing strains, particularly for individuals at risk of D-lactic acidosis, such as patients with short bowel syndrome or carbohydrate malabsorption [52,53]. However, it is essential to note that these bacteria are commonly found in various food sources, including yogurt and other fermented products, which have been safely consumed for generations. Moreover, several *Lactiplantibacillus* probiotics have been classified as “generally recognized as safe” (GRAS) by the United States Food and Drug Administration (US FDA) [54]. Therefore, we suggest that *Lp. pentosus* TBRC 20328 presents a low risk and does not raise safety concerns regarding the production of D-lactic acid.

### 3.6. Biosynthesis Gene Clusters for Bacteriocin and Secondary Metabolite Production

Biosynthesis gene clusters (BGCs) typically consist of two or more components within a genome, each contributing to the production of specialized metabolites and chemical variants. These components include (i) backbone enzymes, which are responsible for the initial step in synthesizing the product, and (ii) tailoring enzymes, which further modify the molecule produced by the backbone enzymes [55]. To achieve a comprehensive identification of secondary metabolite BGCs, we conducted a combined analysis using BAGEL 4, AntiSMASH (v.7.1.0), and PRISM 4. These tools employ distinct databases and detection algorithms, offering complementary strengths that enhance the robustness of BGC prediction and reduce the likelihood of missing clusters of interest. The BAGEL 4 software [33] was used to identify potential bacteriocin clusters and genes associated with antimicrobial protein biosynthesis. The result shows two areas of interest (AOI) in the genome of *Lp. pentosus* TBRC 20328 containing bacteriocin-coding genes that were plantaricin EF and pediocin. Additionally, the AntiSMASH tool [31] was utilized to identify gene clusters responsible for the biosynthesis of secondary metabolites, including non-ribosomal peptide synthetases (NRPs), polyketide synthases (PKSs), ribosomally synthesized and post-translationally modified peptides (RiPPs), and other antimicrobial synthases. Using the AntiSMASH pipeline with the “strict” parameter, no gene clusters were identified. However, when the “relaxed” parameter was applied, two genomic regions of interest were identified: one corresponds to a class II bacteriocin gene encoding plantaricin EF, and the other is involved in the biosynthesis gene cluster of type 3 polyketide synthase (T3PKS).

**Figure 5:**
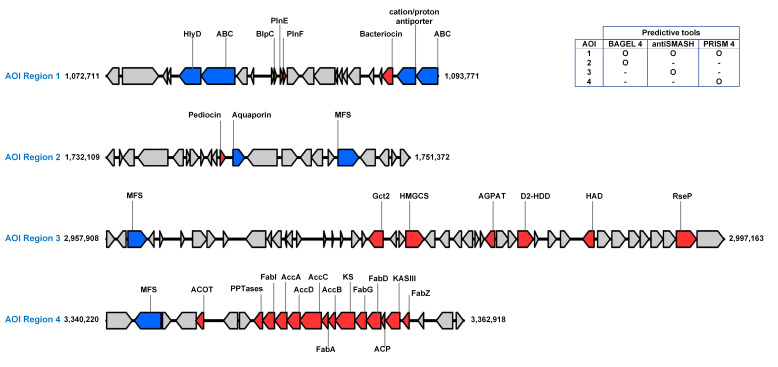
Illustration of four biosynthetic gene clusters in the *Lp. pentosus* TBRC 20328 genome, identified using three predictive tools: anti-SMASH, PRISM4, and BAGEL4. Biosynthetic genes are indicated by red arrows, transporter-related genes by blue arrows, and all other genes by gray arrows. AOI regions 1–4 correspond to the plantaricin EF gene cluster, pediocin gene cluster, type 3 polyketide synthase (T3PKS) gene cluster, and polyketide synthase (PKS) gene cluster, respectively.

Furthermore, the PRISM 4 algorithm [32] was utilized to identify and compare gene clusters for secondary metabolites in the *Lp. pentosus* TBRC 20328 genome. This analysis revealed two antimicrobial gene clusters: one encoding a class II bacteriocin (plantaricin EF) and the other associated with polyketide biosynthesis. Conclusively, the combinatorial exploitation of BAGEL 4 [33], antiSMASH [31], and PRISM 4 [32] led to the identification of four AOIs in the *Lp. pentosus* TBRC 20328 genome, each associated with different metabolites, as illustrated in Figure 5.

AOI region 1, spanning approximately 20 kb, was identified as the genomic region encoding the gene cluster responsible for plantaricin E and plantaricin F biosynthesis in the TBRC 20328 genome, which was consistently generated by all three computational tools. This gene cluster was associated with the biosynthesis of plantaricin EF (PlnEF), a type II bacteriocin comprising two functionally complementary peptides, PlnE and PlnF. To further investigate this gene cluster, we conducted a comparative genomic analysis of the *Lp. pentosus* TBRC 20328 genome alongside 32 other *Lactiplantibacillus* genomes, encompassing 7 *Lp. paraplantarum*, 12 *Lp. pentosus*, and 13 *Lp. plantarum* strains. The result reveals that the PlnEF-encoding gene sequences were highly conserved among the *Lactiplantibacillus* strains studied, which occupy diverse ecological niches. It has been reported that type II bacteriocin is found in LAB, which has antimicrobial activity against pathogenic bacteria [56,57].

The plantaricin EF gene cluster of the strain TBRC 20328 shared high similarity with the corresponding cluster in *Lp. pentosus* strain 68-1, exhibiting 99% sequence identity and 100% coverage. However, the strain 68-1 does not produce these bacteriocins [58]. Notably, there might be genetic discrimination in regulating or expressing bacteriocin gene clusters of different strains of *Lp. pentosus*. Further elucidation of these clusters governing genetic and regulatory mechanisms is necessary to comprehensively understand bacteriocin diversity and function in *Lp. pentosus* TBRC 20328. The existence of antimicrobial gene clusters suggests their ability to produce antimicrobial peptides with enhanced efficacy, positioning the TBRC 20328 strain as a promising candidate for developing natural alternatives to chemical preservatives and antibiotics [59]. Such insights may also inform broader applications of bacteriocins in food technology and the strategic development of natural antimicrobial agents.

The AOI region 2 was predicted in the genome of strain TBRC 20328 by using BAGEL 4, which corresponded to the pediocin-encoding gene, a type of class II bacteriocin gene cluster. A pediocin gene cluster typically consists of four open reading frames (ORFs) encoding the structural peptide, immunity protein, accessory protein, and ABC transporter [60]. A subsequent manual BLAST search at the amino acid level against UniProtKB and NCBI revealed that the putative pediocin gene of the TBRC 20328 strain was similar to the pediocin PA-1 immunity protein. No actual pediocin structural gene was detected within the predicted pediocin gene cluster in this genome.

In Figure 5, the AOI region 3 in the *Lp. pentosus* TBRC 20328 was identified using the antiSMASH tool. This cluster was the largest AOI, comprising 46 genes, which were classified as the Type III Polyketide Synthase (T3PKS) family. T3PKS proteins are typically small, dimeric molecules with a molecular weight between 80 and 90 kDa. These enzymes are involved in the production of polyketides, complex secondary metabolites that serve as the backbone for various bioactive substances, such as antibiotics, antifungals, parasiticides, and immunomodulators [61]. Type III PKSs are among the most common biosynthetic gene clusters found in LAB, indicating their widespread role in secondary metabolism across these microorganisms [62,63]. The presence of the T3PKS gene cluster in *Lp. pentosus* TBRC 20328 suggests that the polyketides synthesized by T3PKS enzymes may contribute to the antimicrobial activity.

Using PRISM 4, the AOI region 4, containing the PKS gene cluster, was identified in the *Lp. pentosus* TBRC 20328 genome. This cluster included a gene encoding an acyltransferase (AT) domain, a crucial component in the synthesis of polyketides. The AT domain is a monomeric unit responsible for producing secondary metabolites. In addition, the analysis revealed several other genes located both upstream and downstream of the AT gene, including additional biosynthetic genes, transporter genes, and the genes encoding key PKS domains, such as ketosynthase (KS) and ketoreductase (KR). The domains of the PKS enzymes of *Lp. pentosus* displayed mechanistic similarities to those of fatty acid synthases (FAS). PKS and FAS share a similar biosynthetic framework, utilizing the same precursors and cofactors in their respective pathways [64,65]. Despite these similarities, the PKS gene cluster of *Lp. pentosus* TBRC 20328 appeared to overlap with gene clusters typically associated with fatty acid synthesis identified through genome annotation, presuming that the pathways for fatty acid and polyketide biosynthesis in this strain may not be as distinctly separated as previously thought. The apparent integration between primary (fatty acids) and secondary (polyketides) metabolic pathways highlights the need for further research to clarify the specific roles and interactions of these biosynthetic pathways in this strain.

The detection of gene clusters associated with bacteriocin and polyketide biosynthesis in *Lp. pentosus* TBRC 20328 postulates its capacity to produce bioactive metabolites with antimicrobial and potentially therapeutic properties. Bacteriocins, ribosomally synthesized antimicrobial peptides produced by LAB, are effective natural preservatives and alternatives to chemical additives in food products [66,67]. Likewise, polyketides are a diverse class of microbial metabolites with well-documented antimicrobial, anti-inflammatory, and antioxidant activities, supporting their use in functional foods and health-promoting formulations [55,68]. These genomic features may support their functional role in modulating microbial communities and enhancing host interactions. From a technological perspective, such genetic traits position the strain as a promising candidate for use in natural preservation and the development of next-generation probiotic and fermented food products, reducing reliance on synthetic additives.

### 3.7. The Phosphotransferase System (PTS) in Carbohydrate Uptake of Lp. pentosus TBRC 20328

The PTS is a complex, multi-component mechanism in bacteria that facilitates the uptake of various sugar substrates, including monosaccharides, disaccharides, amino sugars, polyols, and other sugar derivatives. This system operates by transporting specific saccharides across the bacterial inner membrane through a series of sequential steps for transferring a phosphate group from phosphoenolpyruvate (PEP) to the incoming sugar [69,70]. The PTS comprises various proteins, including soluble phosphotransferases and an integral membrane protein that directly mediates the translocation of sugars into the cytoplasm [71]. Of LAB, particularly the *Lactiplantibacillus* genus, PTS transporters are the primary pathways for carbohydrate transport [72]. Previous studies revealed notable diversity in PTS transporter proteins among different strains; for example, *Lp. plantarum* WCSF1, which harbors 25 PTS transporters [73], *Lp. pentosus* strain 68-1, containing 46 PTS transporters [58], and *Lp. plantarum* MC5, possessing 61 distinct PTS transporters [74]. The variation in PTS transporter abundance strongly correlates with the ability of these strains to utilize diverse sugar substrates, highlighting their metabolic flexibility.

Based on homology with annotated PTS protein-encoding genes, 65 PTS-type sugar transporter proteins associated with various sugars were functionally annotated in the *Lp. pentosus* TBRC 20328 genome (Supplementary Data Table S2). These included six lactose/cellobiose family transporters, six beta-glucosidase transporters, five glucose transporters, four fructose transporters, four galactitol transporters, and four glucitol/sorbitol transporters. It also possessed other transporters for galactosamine (3 transporters), mannose/fructose/sorbose family (3 transporters), sucrose (2 transporters), trehalose (2 transporters), mannitol (1 transporter), N-acetylglucosamine (1 transporter), and 17 unspecified sugar transporters. These findings suggest that *Lp. pentosus* TBRC 20328 could utilize a broad range of carbon sources for cell growth and function, which helps develop culture conditions for specific applications. Additionally, several genes associated with the oligosaccharide phosphate transfer system were found in multiple copies in the TBRC 20328 genome. This genetic redundancy could enhance its capability to utilize these carbon sources efficiently. These functional proteins may confer metabolic flexibility, allowing *Lp. pentosus* TBRC 20328 to thrive and survive in diverse and complex environments, which helps design fermentation processes for functional food production.

### 3.8. Short-Chain Fatty Acid Biosynthesis Genes in Lp. pentosus TBRC 20328

Some LABs have been identified for their capacity to produce SCFAs [75,76]. The investigation of SCFA production by LAB is of particular interest due to its potential health benefits for human hosts [77,78]. Through a functional analysis of the *Lp. pentosus* TBRC 20328 genome, we identified four genes that significantly contribute to acetate production, two genes involved in formate synthesis, and six genes associated with lactate biosynthesis. Our analysis also revealed two thioesterase genes, which may be linked to SCFA production. Previous studies demonstrated that *E. coli* can produce butyrate by expressing heterologous thioesterases, suggesting the potential of these identified genes for heterologous production of butyrate and other SCFAs in a microbial host system of interest [79,80]. However, further investigation is needed to elucidate the functional roles of the thioesterase genes in *Lp. pentosus* TBRC 20328 and their potential contributions to SCFA biosynthetic pathways. Putative genes and enzymes involved in SCFA and lactate synthesis are listed in Supplementary Data Table S3.

The presence of SCFA biosynthetic genes in *Lp. pentosus* TBRC 20328 suggests its potential role in promoting gut microbial balance and supporting host metabolic functions. These metabolites are known to influence intestinal barrier maintenance and immune modulation [81,82]. From an industrial innovation standpoint, such genetic capabilities position the strain as a valuable ingredient for next-generation functional foods and probiotic formulations that target digestive and systemic health, offering a naturally derived alternative to conventional supplements.

### 3.9. Exopolysaccharides (EPS) Gene Clusters in Lp. pentosus TBRC 20328

To investigate the genes related to the EPS production, we performed a comparative whole-genome analysis of *Lp. pentosus* TBRC 20328 and other previously characterized *Lactiplantibacillus* strains [74] downloaded from the NCBI database. The selected strains, including *Lp. plantarum* WCFS1, ST-III, JDM1, and SMB758, were chosen for this study due to the confirmed presence of *eps* genes in their genomes and the extensive characterization of their gene clusters. *Lp. plantarum subsp. plantarum* ST-III is an outlier, harboring three complete *eps* gene clusters: *eps2ABCEF*, *eps3ABDEFHIJ*, and *eps4ABCEFGHIJ*. Our analysis revealed that the genome of *Lp. pentosus* TBRC 20328 also contained three *eps* gene clusters, showing high similarity to those identified in *Lp. plantarum* ST-III. Furthermore, A distinct *eps*F gene was identified which shared a high degree of sequence similarity with the corresponding genes found in *Lp. plantarum* JDM1 and SMB758 (Table 3). However, we did not find any *eps* gene similarity between our strain and *Lp. plantarum* WCFS1. Cluster 3 of the *eps* genes in *Lp. pentosus* TBRC 20328 notably encoded a *wzx* gene, which plays a critical role in the transport of glycosyl units during exopolysaccharide (EPS) biosynthesis [83]. The presence of this gene indicates a strong capacity for repeat unit transport within this cluster. Based on genomic similarity, we propose that TBRC 20328 is a close genetic relative of *Lp. plantarum subsp. plantarum* ST-III. The presence of multiple eps gene clusters in both strains may contribute to their enhanced EPS production capabilities.

EPSs produced by *Lactiplantibacillus* species are increasingly recognized for their diverse health-promoting properties. Studies have shown that LAB-derived EPSs can exhibit immunomodulatory activity [84], enhance gut barrier integrity [85], and promote the growth of beneficial gut microbiota [11]. Additionally, EPSs may contribute to antioxidant effects [86], cholesterol-lowering potential [87], and inhibition of pathogen adhesion in the gastrointestinal tract [88]. The genomic features observed in *Lp. pentosus* TBRC 20328 suggest its potential to produce functionally relevant EPSs with similar bioactivities. However, it is important to note that these conclusions are based solely on in silico analysis, and further experimental validation is required to confirm the strain’s EPS production capacity and its associated health benefits.

### 3.10. Comparative Analysis of Lactiplantibacillus Genomes Exploring Unique Genetic Features of Lp. pentosus Strains

A comparative genomics analysis was performed across 33 *Lactiplantibacillus*, including 13 *Lp. pentosus*, 13 *Lp. plantarum*, and 7 *Lp. paraplantarum*, using an all-against-all genome comparison approach with OrthoFinder [34]. This analysis identified orthologous genes and orthogroups, thoroughly characterizing the genomic relationships, core genome, and species-specific content of *Lp. pentosus* strains. A comparative study of 13 *Lp. pentosus* genomes identified 2380 orthogroups of orthologous protein-coding genes, collectively referred to as the “consensus *Lp. pentosus* gene set”. Notably, variation in copy number was observed across these orthogroups, suggesting strain-specific genomic divergence within *Lp. pentosus* strains. Further analysis revealed 1880 orthogroups (78.99%) of the consensus *Lp. pentosus* gene set were conserved across the 33 *Lactiplantibacillus* genomes and were thus classified as the core gene set shared among these species. In contrast, 94 orthogroups (out of the identified 2380 orthogroups) were exclusive to the 13 *Lp. pentosus* genomes and were absent in the *Lp. plantarum* and *Lp. paraplantarum* genomes analyzed in this study. These proteins were designated “specific orthogroups” for *Lp. pentosus*. Based on genome annotation data of *Lp. pentosus* strain TBRC 20328, these specific orthogroups were classified into five functional categories: (i) protein-coding genes involved in metabolism (17 of total specific orthogroups), (ii) toxin–antitoxin system proteins (7 of total specific orthogroups), (iii) transcriptional regulators (6 of total specific orthogroups), (iv) transporter proteins (15 of total specific orthogroups), and (v) unclassified functions (49 of total specific orthogroups) (Supplementary Data Table S4). Among these, we focused on the protein-coding genes related to metabolism, which are likely critical to the species’ ecological role and metabolic adaptability.

One of the most notable findings was the discovery of the malonate decarboxylase (mdc) gene cluster, a unique genetic feature present exclusively in *Lp. pentosus* and absent in both *Lp. plantarum* and *Lp. paraplantarum*. The *mdc* gene cluster in *Lp. pentosus* TBRC 20328 consists of five distinct subunits: alpha (α), beta (β), gamma (γ), delta (δ), and epsilon (ε), which work together to form a functional enzymatic complex responsible for malonate decarboxylation, a process critical for carbon metabolism. In addition, we identified a LysR-family transcriptional regulator (LTTR) associated with the mdc operon. This regulator plays a crucial role in activating transcription of the gene cluster, a mechanism previously described for *Lp. pentosus* strain KCA1 [89], corresponding to tightly regulated expression of the malonate decarboxylase system in *Lp. pentosus* [90,91].

Malonate decarboxylase catalyzes the decarboxylation of malonate to acetate and CO_2_, a reaction that is driven cyclically by acetyl-CoA. The α, β, and γ subunits are central to this process. In contrast, the δ and ε subunits function as an acyl-carrier protein (ACP) and a malonyl-CoA processing protein, respectively [92,93]. Our findings suggest that *Lp. pentosus* TBRC 20328 might be capable of utilizing malonate as a sole carbon source for growth and metabolic energy by incorporating the malonate decarboxylase system as a key component of its metabolic pathways, similar to several bacterial species [93].

Additionally, we identified four genes involved in the leucine biosynthesis pathway in *Lp. pentosus* TBRC 20328, including 2-isopropylmalate synthase (*leuA*), 3-isopropylmalate dehydrogenase (*leuB*), 3-isopropylmalate dehydratase large subunit (*leuC*), and 3-isopropylmalate dehydratase small subunit (*leuD*). These enzymes convert precursor molecules into leucine, an essential amino acid. These genes were found to be present exclusively in *Lp. pentosus*, which is consistent with the previous reports on other *Lp. pentosus* strains, including IG1 [94] and KCA1 [89], suggesting that there is a conserved mechanism for leucine biosynthesis in *Lp. pentosus* that might offer metabolic advantages related to amino acid production and ecological adaptability.

Furthermore, we identified an additional copy of the acetyl-CoA carboxylase (ACC) in *Lp. pentosus* compared to *Lp. plantarum* and *Lp. paraplantarum*. This extra gene copy included two components: the acetyl-CoA carboxylase biotin carboxyl carrier protein subunit (BCCP) and the acetyl-CoA carboxylase biotin carboxylase subunit (BC). ACCs are key enzymes that catalyze the conversion of acetyl-CoA to malonyl-CoA, a crucial intermediate in fatty acid biosynthesis and autotrophic carbon fixation. This process occurs in two steps: First, biotin is carboxylated in an ATP-dependent reaction catalyzed by the biotin carboxylase subunit of ACC. In the second step, the carboxyl transferase subunit of ACC transfers bicarbonate to acetyl-CoA, forming malonyl-CoA [95]. The additional ACC gene copy found in *Lp. pentosus* indicated that such metabolic evolution has advantages for growth and survival adaptation, particularly in lipid synthesis and energy metabolism processes.

**Figure 6:**
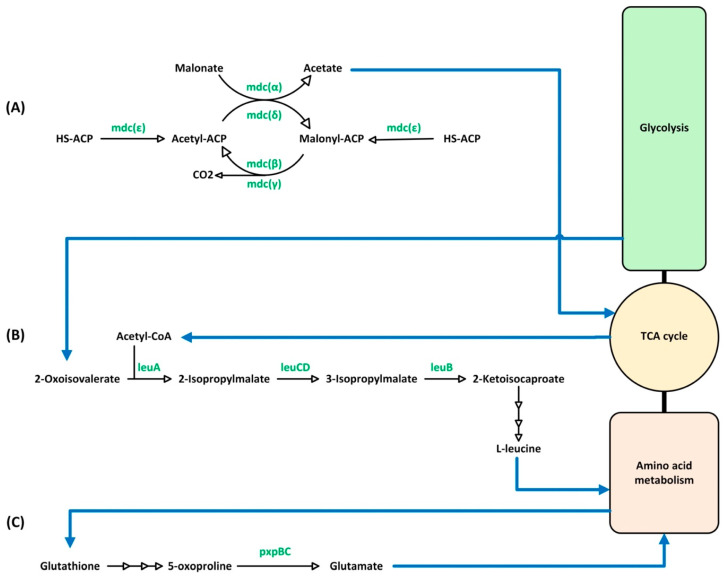
Overview of *Lp. pentosus*-specific pathways. (**A**) Malonate decarboxylase system, which catalyzes the decarboxylation of malonate to acetate, playing a role in energy metabolism; (**B**) leucine biosynthesis pathway, essential for the synthesis of the branched-chain amino acid leucine; and (**C**) the oxoprolinase system, involved in the metabolism of 5-oxoproline, contributing to amino acid turnover and glutathione metabolism. Blue arrows indicate the direction of metabolic flow.

Finally, our comparative genomic analysis revealed that putative genes associated with the γ-glutamyl cycle were uniquely present in *Lp. pentosus* but absent in *Lp. plantarum* and *Lp. paraplantarum*. 5-Oxoprolinase catalyzes the ATP-dependent conversion of 5-oxoproline to L-glutamate, a crucial step in the γ-glutamyl cycle, typically found in eukaryotes [96]. Although most prokaryotes lack homologs for this enzyme and do not exhibit the γ-glutamyl cycle [97], the previous studies linked prokaryotic 5-oxoprolinase to a conserved gene cluster comprising pxpA, pxpB, and pxpC. In this study, we identified only two conserved genes in the 5-oxoprolinase cluster of *Lp. pentosus* TBRC 20328, including the gene encoding 5-oxoprolinase subunit B (pxpB) and a biotin-dependent carboxyltransferase family protein (pxpC). These findings suggest that a unique gene composition of the 5-oxoprolinase cluster existed in *Lp. pentosus*, and further experimental investigation is required to elucidate the functional roles of subunits B and C in this species. A summary of the species-specific pathways of *Lp. pentosus* is illustrated in Figure 6.

## 4. Conclusions

This study presents comprehensive genomic insights into the safety and functional potential of *Lp. pentosus* TBRC 20328, supporting its application in food and biotechnological industries. The absence of antimicrobial resistance genes, virulence factors, and other undesirable genetic elements confirms its compliance with international safety standards when used under recommended conditions. The presence of genes encoding bacteriocins and PKS underscores its potential for the development of natural preservatives, functional food ingredients, and probiotic formulations. Comparative genomic analysis also revealed a metabolically versatile profile, including pathways for sugar transport, malonate decarboxylation, and leucine biosynthesis traits that contribute to its adaptability and efficiency in diverse fermentation environments. These characteristics make *Lp. pentosus* TBRC 20328 a promising candidate for large-scale probiotic production and the creation of innovative dietary supplements. Overall, this work provides a strong foundation for future omics- and AI-guided studies and highlights the strain’s potential as a chassis for genetic engineering and precision fermentation in industrial applications.

## Figures and Tables

**Table 1:** Genome statistics of *Lactiplantibacillus* TBRC 20328.

	Genomic	Plasmid A	Plasmid B	Plasmid C	Plasmid D	Plasmid E	Total
Total base (bp)	3,491,384	70,426	68,335	48,057	55,487	6885	3,740,574
A (bp)	932,443	20,620	20,892	15,051	14,869	2133	1,006,008
T (bp)	932,054	22,020	19,008	14,340	17,651	2112	1,007,185
G (bp)	813,600	13,556	14,549	9448	11,106	1306	863,565
C (bp)	813,287	14,230	13,886	9218	11,861	1334	863,816
GC content (%)	46.60	39.45	41.61	38.84	41.39	38.34	46.18
No. of protein-coding genes	3056	79	81	54	62	8	3340
Avg. gene length (bp)	919	708	691	649	798	328	900
No. of tRNA genes	63	-	-	-	-	-	63
No. of tmRNA genes	1	-	-	-	-	-	1
No. of rRNA genes	16	-	-	-	-	-	16
Repeat regions	3	-	-	-	-	-	3

**Table 2:** Plasmids in *Lactiplantibacillus* TBRC 20328 searched by PlasmidFinder.

Plasmid	Start	End	PlasmidFinder Annotation	Origin	Identity (%)
A	44,904	45,785	rep38_2_repA (LBPp1)	*Lp. plantarum* P-8	96.19
D	21,409	22,435	rep38_1_rep (pLBUC03)	*L. buchneri* NRRL B-30929	86.50

**Table 3:** The *eps* gene clusters and their homologous sequences in *Lp. pentosus* TBRC 20328.

GeneID	Length (aa)	NCBI Accession	Species	Length (aa)	Gene	Gene Function	Homology (%)
*EPS* gene Cluster 1							
Orf00270	376	ADN98210	*Lp. plantarum* ST-III	359	*eps3I*	O-acetyltransferase	267/360 (74%)
Orf00271	367	ADN98209	*Lp. plantarum* ST-III	369	*eps3H*	polysaccharide biosynthesis protein	287/367 (78%)
Orf00272	393	ADN98208	*Lp. plantarum* ST-III	406	*eps3F*	polysaccharide polymerase	301/388 (78%)
Orf00273	207	ADN98207	*Lp. plantarum* ST-III	210	*eps3E*	polysaccharide biosynthesis protein	141/207 (68%)
Orf00274	375	ADN98206	*Lp. plantarum* ST-III	377	*eps3D*	polysaccharide biosynthesis protein	248/375 (66%)
Orf00276	310	ADN98204	*Lp. plantarum* ST-III	310	*eps3B*	glycosyltransferase	308/310 (99%)
Orf00277	303	ADN98203	*Lp. plantarum* ST-III	303	*eps3A*	glycosyltransferase	294/303 (97%)
*EPS gene Cluster * 2							
Orf00292	225	ADN98183	*Lp. plantarum* ST-III	200	*eps2E*	priming glycosyltransferase	200/200 (100%)
Orf00294	271	ADN98181	*Lp. plantarum* ST-III	257	*eps2C*	exopolysaccharide biosynthesis protein	252/257 (98%)
Orf00295	242	ADN98180	*Lp. plantarum* ST-III	242	*eps2B*	exopolysaccharide biosynthesis protein	241/242 (99%)
Orf00296	256	ADN98179	*Lp. plantarum* ST-III	256	*eps2A*	exopolysaccharide biosynthesis protein	254/256 (99%)
*EPS *gene Cluster 3							
Orf00286	460	ADN98188	*Lp. plantarum* ST-III	460	*wxz*	exopolysacharide protein Wzx	451/460 (98%)
Orf00288	319	ADN98187	*Lp. plantarum* ST-III	319	*eps4I*	glycosyltransferase	314/319 (98%)
Orf00289	424	ADN98186	*Lp. plantarum* ST-III	424	*eps4H*	polysaccharide polymerase	421/424 (99%)
Orf00290	343	ADN98185	*Lp. plantarum* ST-III	345	*eps4G*	glycosyltransferase	341/343 (99%)
Orf00291	364	ADN98184	*Lp. plantarum* ST-III	364	*eps4F*	glycosyltransferase	359/364 (99%)
Orf02621	251	ADN98956	*Lp. plantarum* ST-III	252	*eps4A*	exopolysaccharide biosynthesis protein	194/253 (77%)
Orf02622	238	ADN98955	*Lp. plantarum* ST-III	235	*eps4B*	capsular exopolysaccharide family protein	193/234 (82%)
		WP_015640554	*Lp. plantarum* JDM1	235	*epsD/* *epsB/* *epsF*	CpsD/CapB family tyrosine-protein kinase	192/234 (82%)
		WDQ20187	*Lp. plantarum* SMB758	235	*epsD/epsB*	CpsD/CapB family tyrosine-protein kinase	193/234 (82%)
Orf02623	259	ADN98954	*Lp. plantarum* ST-III	273	*eps4C*	phosphotyrosine-protein phosphatase	184/259 (71%)
Orf02625	221	ADN98952	*Lp. plantarum* ST-III	223	*eps4E*	priming glycosyltransferase	171/221 (77%)
Orf02630	481	ADN98947	*Lp. plantarum* ST-III	483	*eps4J*	repeat unit transporter	353/481 (73%)
Specific *EPS* gene							
Orf00813	245	WP_003643865	*Lp. plantarum* JDM1	245	*epsF*	WecB/TagA/CpsF family glycosyltransferase	225/245 (92%)
		WDQ21455	*Lp. plantarum* SMB758	245	*epsF*	WecB/TagA/CpsF family glycosyltransferase	225/245 (92%)

## Data Availability

The data presented in this study are openly available in the NCBI Sequence Read Archive, with the accession numbers CP191162-CP191167.

## Supplementary Materials

The following supporting information can be downloaded at: https://www.mdpi.com/article/10.3390/foods14172973/s1, Table S1: Assessment of genome completeness.; Table S2. Sugar-specific phosphate transport system in *Lp. pentosus* TBRC 20328. Table S3: Genes involved in short-chain fatty acid biosynthesis identified in the *Lp. pentosus* TBRC 20328 genome. Table S4: List of genes in *Lp. pentosus* TBRC 20328 identified as part of orthogroups unique to the *Lp. pentosus* group.

## Abbreviations

The following abbreviations are used in this manuscript:

ACC	Acetyl-CoA carboxylase
ACP	Acyl-carrier protein
AMR	Antimicrobial resistance
ANI	Average nucleotide identity
AOI	Area of interest
ARG	Antibiotic resistance gene
AT	Acyltransferase
BA	Biogenic amine
BC	Acetyl-CoA carboxylase biotin carboxylase subunit
BCCP	Biotin carboxyl carrier protein subunit
BGC	Biosynthesis gene cluster
BSH	Bile salt hydrolase
COG	Clusters of Orthologous Group
EPS FAS	Exopolysaccharide Fatty acid synthase
KS	Ketosynthase
KR	Ketoreductase
LAB	Lactic acid bacteria
Lar	Lactate racemase
LDHD	D-lactate dehydrogenase
LTTR	LysR-family transcriptional regulator
PEP	Phosphoenolpyruvate
PKS	Polyketide synthase
PTS	Phosphotransferase system
RiPP	Ribosomally synthesized and post-translationally modified peptides
SCFA	Short-chain fatty acid
VF	Virulence factor

## References

[1] Garcia-Gonzalez N., Battista N., Prete R., Corsetti A. (2021). Health-promoting role of *Lactiplantibacillus plantarum* isolated from fermented foods. Microorganisms.

[2] Yilmaz B., Bangar S.P., Echegaray N., Suri S., Tomasevic I., Manuel Lorenzo J., Melekoglu E., Rocha J.M., Ozogul F. (2022). The impacts of *Lactiplantibacillus plantarum* on the functional properties of fermented foods, A review of current knowledge. Microorganisms.

[3] Ricciardi A., Parente E., Guidone A., Ianniello R.G., Zotta T., Abu Sayem S., Varcamonti M. (2012). Genotypic diversity of stress response in *Lactobacillus plantarum*, *Lactobacillus paraplantarum* and *Lactobacillus pentosus*. Int. J. Food Microbiol..

[4] Chaudhary A., Saharan B.S. (2019). Probiotic properties of *Lactobacillus plantarum*. J. Pure Appl. Microbiol..

[5] Huang J.Y., Kao C.Y., Liu W.S., Fang T.J. (2017). Characterization of high exopolysaccharide-producing *Lactobacillus* strains isolated from mustard pickles for potential probiotic applications. Int. Microbiol..

[6] Sionek B., Szydłowska A., Küçükgöz K., Kołożyn-Krajewska D. (2023). Traditional and new microorganisms in lactic acid fermentation of food. Fermentation.

[7] Lee Y.S., Kim M.J., Park S.H. (2020). Biotechnological potential of *Lactiplantibacillus pentosus* in food fermentation and preservation. J. Food Sci..

[8] Pimentel T.C., Almeida D., Mota M. (2021). Probiotic and functional properties of *Lactiplantibacillus pentosus*, Applications in food and health. Probiotics Antimicro..

[9] Gänzle M.G., Follador R., Hertel C. (2016). Lactic acid bacteria in fermented foods. Food Res. Int..

[10] Hasan H.A., Rahim N.F.M., Alias J., Ahmad J., Said N.S.M., Ramli N.N., Buhari J., Abdullah S.R.S., Othman A.R., Jusoh H.H.W. (2024). A Review on the roles of extracellular polymeric substances (EPSs) in wastewater treatment: Source, mechanism study, bioproducts, limitations, and future challenges. Water.

[11] Wang W., Ju Y., Liu N., Shi S., Hao L. (2023). Structural characteristics of microbial exopolysaccharides in association with their biological activities: A review. Chem. Biol. Technol. Agric..

[12] Gao Y., Li D. (2018). Screening of lactic acid bacteria with cholesterol-lowering and triglyceride-lowering activity in vitro and evaluation of probiotic function. Ann. Microbiol..

[13] Rocha-Ramírez L.M., Pérez-Solano R.A., Castañón-Alonso S.L., Moreno Guerrero S.S., Pacheco A.R., Garibay M.G., Eslava C. (2017). Probiotic *Lactobacillus* strains stimulate the inflammatory response and activate human macrophages. J. Immunol. Res..

[14] Magaldi S., Mata-Essayag S., Hartung de Capriles C., Perez C., Colella M.T., Olaizola C., Ontiveros Y. (2004). Well diffusion for antifungal susceptibility testing. Int. J. Infect. Dis..

[15] Chin C.S., Alexander D., Marks P., Klammer A.A., Drake J., Heiner C., Clum A., Copeland A., Huddleston J., Eichler E.E. (2013). Nonhybrid, finished microbial genome assemblies from long-read SMRT sequencing data. Nat. Methods.

[16] Seeman T. (2014). Prokka: Rapid prokaryotic genome annotation. Bioinformatics.

[17] Cantalapiedra C.P., Hernández-Plaza A., Letunic I., Bork P., Huerta-Cepas J. (2021). eggNOG-mapper v2, functional annotation, orthology assignments, and domain prediction at the metagenomic scale. Mol. Biol. Evol..

[18] Huerta-Cepas J., Szklarczyk D., Heller D., Hernández-Plaza A., Forslund S.K., Cook H., Mende D.R., Letunic I., Rattei T., Jensen L.J. (2019). eggNOG 5.0, a hierarchical, functionally and phylogenetically annotated orthology resource based on 5090 organisms and 2502 viruses. Nucleic Acids Res..

[19] Buchfink B., Xie C., Huson D. (2015). Fast and sensitive protein alignment using DIAMOND. Nat. Methods.

[20] Tamura K., Stecher G., Kumar S. (2021). MEGA11: Molecular Evolutionary Genetics Analysis version 11. Mol. Biol. Evol..

[21] Lee I., Ouk Kim Y., Park S.C., Chun J. (2016). OrthoANI: An improved algorithm and software for calculating average nucleotide identity. Int. J. Syst. Evol. Microbiol..

[22] Richter M., Rosselló-Móra R. (2009). Shifting the genomic gold standard for the prokaryotic species definition. Proc. Natl. Acad. Sci. USA.

[23] Alcock B.P., Huynh W., Chalil R., Smith K.W., Raphenya A.R., Wlodarski M.A., Edalatmand A., Petkau A., A Syed S., Tsang K.K. (2023). CARD 2023: Expanded curation, support for machine learning, and resistome prediction at the Comprehensive Antibiotic Resistance Database. Nucleic Acids Res..

[24] Gupta S.K., Padmanabhan B.R., Diene S.M., Lopez-Rojas R., Kempf M., Landraud L., Rolain J.M. (2014). ARG-ANNOT, a new bioinformatic tool to discover antibiotic resistance genes in bacterial genomes. Antimicrob. Agents Chemother..

[25] Liu B., Zheng D., Zhou S., Chen L., Yang J. (2022). VFDB 2022, a general classification scheme for bacterial virulence factors. Nucleic Acids Res..

[26] Bortolaia V., Kaas R.F., Ruppe E., Roberts M.C., Schwarz S., Cattoir V., Philippon A., Allesoe R.L., Rebelo A.R., Florensa A.F. (2020). ResFinder 4.0 for predictions of phenotypes from genotypes. J. Antimicrobl. Chemother..

[27] Doster E., Lakin S.M., Dean C.J., Wolfe C., Young J.G., Boucher C., E Belk K., Noyes N.R., Morley P.S. (2020). MEGARes 2.0, a database for classification of antimicrobial drug, biocide and metal resistance determinants in metagenomic sequence data. Nucleic Acids Res..

[28] Feldgarden M., Brover V., Haft D.H., Prasad A.B., Slotta D.J., Tolstoy I., Tyson G.H., Zhao S., Hsu C.-H., McDermott P.F. (2019). Validating the AMRFinder tool and resistance gene database by using antimicrobial resistance genotype-phenotype correlations in a collection of isolates. Antimicrob. Agents Chemother..

[29] Carattoli A., Zankari E., García-Fernández A., Larsen M.V., Lund O., Voldby Villa L., Møller Aarestrup F., Hasman H. (2014). In silico detection and typing of plasmids using PlasmidFinder and plasmid multilocus sequence typing. Antimicrob. Agents Chemother..

[30] Seemann T. ABRicate. Github. https://github.com/tseemann/abricate.

[31] Blin K., Shaw S., Augustijn H.E., Reitz Z.L., Biermann F., Alanjary M., Fetter A., Terlouw B.R., Metcalf W.W., Helfrich E.J.N. (2023). antiSMASH 7.0: New and improved predictions for detection, regulation, chemical structures and visualization. Nucleic Acids Res..

[32] Skinnider M.A., Johnston C.W., Gunabalasingam M., Merwin N.J., Kieliszek A.M., MacLellan R.J., Li H., Ranieri M.R.M., Webster A.L.H., Cao M.P.T. (2020). Comprehensive prediction of secondary metabolite structure and biological activity from microbial genome sequences. Nat. Commun..

[33] van Heel A.J., de Jong A., Song C., Viel J.H., Kok J., Kuipers O.P. (2018). BAGEL4, a user-friendly web server to thoroughly mine RiPPs and bacteriocins. Nucleic Acids Res..

[34] Emms D.M., Kelly S. (2019). OrthoFinder, phylogenetic orthology inference for comparative genomics. Genome Biol..

[35] Manni M., Berkeley M.R., Seppey M., Simão F.A., Zdobnov E.M. (2021). BUSCO Update, Novel and streamlined workflows along with broader and deeper phylogenetic coverage for scoring of eukaryotic, prokaryotic, and viral genomes. Mol. Biol. Evol..

[36] Nishimura O., Hara Y., Kuraku S. (2019). Evaluating genome assemblies and gene models using gVolante. Methods Mol. Biol..

[37] Park D.H., Imelfort M., Skennerton C.T., Hugenholtz P., Tyson G.W. (2015). CheckM: Assessing the quality of microbial genomes recovered from isolates, single cells, and metagenomes. Genome Res..

[38] Galperin M.Y., Wolf Y.I., Makarova K.S., Alvarez R.V., Landsman D., Koonin E.V. (2021). COG database update, focus on microbial diversity, model organisms, and widespread pathogens. Nucleic Acids Res..

[39] Kanehisa M., Furumichi M., Sato Y., Kawashima M., Ishiguro-Watanabe M. (2023). KEGG for taxonomy-based analysis of pathways and genomes. Nucleic Acids Res..

[40] Cumsille A., Durán R.E., Rodríguez-Delherbe A., Saona-Urmeneta V., Cámara B., Seeger M., Araya M., Jara N., Buil-Aranda C., Ioshikhes I. (2023). GenoVi, an open-source automated circular genome visualizer for bacteria and archaea. PLoS Comput. Biol..

[41] Chun J., Oren A., Ventosa A., Christensen H., Arahal D.R., da Costa M.S., Rooney A.P., Yi H., Xu X.-W., De Meyer S. (2018). Proposed minimal standards for the use of genome data for the taxonomy of prokaryotes. Int. J. Syst. Evol. Microbiol..

[42] Wishart D.S., Han S., Saha S., Oler E., Peters H., Grant J.R., Stothard P., Gautam V. (2023). PHASTEST, faster than PHASTER, better than PHAST. Nucleic Acids Res..

[43] Li L., Dechesne A., Madsen J.S., Nesme J., Sorensen S.J., Smets B.F. (2020). Plasmids persist in a microbial community by providing fitness benefit to multiple phylotypes. ISME J..

[44] Barbieri F., Montanari C., Gardini F., Tabanelli G. (2019). Biogenic amine production by lactic acid bacteria, a review. Foods.

[45] Turna N.S., Chung R., McIntyre L. (2024). A review of biogenic amines in fermented foods, occurrence and health effects. Heliyon.

[46] Chokesajjawatee N., Santiyanont P., Chantarasakha K., Kocharin K., Thammarongtham C., Lertampaiporn S., Vorapreeda T., Srisuk T., Wongsurawat T., Jenjaroenpun P. (2020). Safety assessment of a Nham starter culture *Lactobacillus plantarum* BCC9546 via whole-genome analysis. Sci. Rep..

[47] Brashears M.M., Gilliland S.E., Buck L.M. (1998). Bile salt deconjugation and cholesterol removal from media by *Lactobacillus casei*. J. Dairy Sci..

[48] Ahn Y.T., Kim G.B., Lim K.S., Baek Y.J., Kim H.U. (2003). Deconjugation of bile salts by *Lactobacillus acidophilus* isolates. Int. Dairy J..

[49] Dong Z., Lee B.H. (2018). Bile salt hydrolases, structure and function, substrate preference, and inhibitor development. Protein Sci..

[50] Prete R., Long S.L., Gallardo A.L., Gahan C.G., Corsetti A., Joyce S.A. (2020). Beneficial bile acid metabolism from *Lactobacillus plantarum* of food origin. Sci. Rep..

[51] Goffin P., Deghorain M., Mainardi J.L., Tytgat I., Champomier-Vergès M.C., Kleerebezem M., Hols P. (2005). Lactate racemization as a rescue pathway for supplying D-lactate to the cell wall biosynthesis machinery in *Lactobacillus plantarum*. J. Bacteriol..

[52] Pohanka M. (2020). D-lactic acid as a metabolite, toxicology, diagnosis, and detection. Biomed. Res. Int..

[53] Remund B., Yilmaz B., Sokollik C. (2023). D-Lactate, Implications for gastrointestinal diseases. Children.

[54] Ricci A., Allende A., Bolton D., Chemaly M., Davies R., Girones R., Koutsoumanis K., Herman L., Lindqvist R., Nørrung B. (2017). Update of the list of QPS-recommended biological agents intentionally added to food or feed as notified to EFSA 5, Suitability of taxonomic units notified to EFSA until September 2016. EFSA J..

[55] Medema M., Kottmann R., Yilmaz P., Cummings M., Biggins J.B., Blin K., de Bruijn I., Chooi Y.H., Claesen J., Coates R.C. (2015). Minimum information about a biosynthetic gene cluster. Nat. Chem. Biol..

[56] Ekblad B., Kyriakou P.L., Oppegard C., Nissen-Meyer J., Kaznessis Y.N., Kristiansen P.E. (2016). Structure-function analysis of the two-peptide bacteriocin plantaricin EF. Biochemistry.

[57] Wang Y., Wei Y., Shang N., Li P. (2022). Synergistic inhibition of plantaricin E/F and lactic acid against *Aeromonas hydrophila* LPL-1 reveals the novel potential of class IIb bacteriocin. Front. Microbiol..

[58] Xue W., Liu C., Liu Y., Ding H., An C., Zhang S., Ma S., Zhang Q. (2024). Probiotic evaluation of *Lactiplantibacillus pentosus* 68-1, a rutin conversion strain isolated from Jiangshui, by genomic analysis and tests In Vitro. Fermentation.

[59] Kareem R.A., Razavi S.H. (2020). Plantaricin bacteriocins, as safe alternative antimicrobial peptides in food preservation—A review. J. Food Saf..

[60] Van Reenen C.A., Chikindas M.L., Van Zyl W.H., Dicks L.M. (2003). Characterization and heterologous expression of a class IIa bacteriocin, plantaricin 423 from *Lactobacillus plantarum* 423, in *Saccharomyces cerevisiae*. Int. J. Food Microbiol..

[61] Lim Y.P., Go M.K., Yew W.S. (2016). Exploiting the biosynthetic potential of type III polyketide synthases. Molecules.

[62] Kumar M.M., Dhanasekaran D. (2021). Biosynthetic gene cluster analysis in *Lactobacillus* species using antiSMASH. Advances in Probiotics.

[63] Okoye C.O., Dong K., Wang Y., Gao L., Li X., Wu Y., Jiang J. (2022). Comparative genomics reveals the organic acid biosynthesis metabolic pathways among five lactic acid bacterial species isolated from fermented vegetables. N. Biotechnol..

[64] Liou G.F., Khosla C. (2003). Building-block selectivity of polyketide synthases. Curr. Opin. Chem. Biol..

[65] West A.R., Bailey C.B. (2023). Crosstalk between primary and secondary metabolism, Interconnected fatty acid and polyketide biosynthesis in prokaryotes. Bioorg. Med. Chem. Lett..

[66] Zacharof M.P., Lovitt R.W. (2012). Bacteriocins produced by lactic acid bacteria a review article. APCBEE Procedia.

[67] Demirgül F., Kaya H.İ., Ucar R.A., Mitaf N.A., Şimşek Ö. (2025). Expanding layers of bacteriocin applications: From food preservation to human health interventions. Fermentation.

[68] Shen B. (2003). Polyketide biosynthesis beyond the type I, II and III polyketide synthase paradigms. Curr. Opin. Chem. Biol..

[69] Kotrba P., Masayuki I., Yukawa H. (2001). Bacterial phosphotransferase system (PTS) in carbohydrate uptake and control of carbon metabolism. J. Biosci. Bioeng..

[70] Deutscher J., Francke C., Postma P.W. (2006). How phosphotransferase system-related protein phosphorylation regulates carbohydrate metabolism in bacteria. Microbiol. Mol. Biol. Rev..

[71] McCoy J.G., Levin E.J., Zhou M. (2015). Structural insight into the PTS sugar transporter EIIC. Biochim. Biophys. Acta..

[72] Cui Y., Wang M., Zheng Y., Miao K., Qu X. (2021). The carbohydrate metabolism of *Lactiplantibacillus plantarum*. Int. J. Mol. Sci..

[73] Kleerebezem M., Boekhorst J., van Kranenburg R., Molenaar D., Kuipers O.P., Leer R., Tarchini R., Peters S.A., Sandbrink H.M., Fiers M.W. (2003). Complete genome sequence of *Lactobacillus plantarum* WCFS1. Proc. Natl. Acad. Sci. USA.

[74] Zhao X., Liang Q., Song X., Zhang Y. (2023). Whole genome sequence of *Lactiplantibacillus plantarum* MC5 and comparative analysis of eps gene clusters. Front. Microbiol..

[75] Thananimit S., Pahumunto N., Teanpaisan R. (2022). Characterization of short chain fatty acids produced by selected potential probiotic *Lactobacillus* strains. Biomolecules.

[76] Fusco W., Lorenzo M.B., Cintoni M., Porcari S., Rinninella E., Kaitsas F., Lener E., Mele M.C., Gasbarrini A., Collado M.C. (2023). Short-chain fatty-acid-producing bacteria, Key components of the human gut microbiota. Nutrients.

[77] Thirabunyanon M., Hongwittayakorn P. (2013). Potential probiotic lactic acid bacteria of human origin induce antiproliferation of colon cancer cells via synergic actions in adhesion to cancer cells and short-chain fatty acid bioproduction. Appl. Biochem. Biotechnol..

[78] LeBlanc J.G., Chain F., Martín R., Bermúdez-Humarán L.G., Courau S., Langella P. (2017). Beneficial effects on host energy metabolism of short-chain fatty acids and vitamins produced by commensal and probiotic bacteria. Microb. Cell Fact..

[79] Jawed K., Mattam A.J., Fatma Z., Wajid S., Abdin M.Z., Yazdani S.S. (2016). Engineered production of short chain fatty acid in *Escherichia coli* using fatty acid synthesis pathway. PLoS ONE.

[80] Zhao C., Dong H., Zhang Y., Li Y. (2019). Discovery of potential genes contributing to the biosynthesis of short-chain fatty acids and lactate in gut microbiota from systematic investigation in *E. coli.* npj Biofilms. Microbiomes.

[81] Parada Venegas D., De la Fuente M.K., Landskron G., González M.J., Quera R., Dijkstra G., Harmsen H.J.M., Faber K.N., Hermoso M.A. (2019). Short chain fatty acids (SCFAs)-mediated gut epithelial and immune regulation and its relevance for inflammatory bowel diseases. Front. Immunol..

[82] Li B., Ye L., Chen Y., Zhang H. (2025). Genomic insights into probiotic metabolism of dietary carbohydrates, proteins, and fats. Curr. Opin. Food Sci..

[83] Xu Y.M., Cui Y.L., Yue F.F., Liu L.H., Shan Y.Y. (2019). Exopolysaccharides produced by lactic acid bacteria and *Bifidobacteria*: Structures, physiochemical functions and applications in the food industry. Food Hydrocoll..

[84] Rahbar Saadat Y., Yari Khosroushahi A., Pourghassem Gargari B. (2019). A comprehensive review of anticancer, immunomodulatory and health beneficial effects of the lactic acid bacteria exopolysaccharides. Carbohydr. Polym..

[85] Thoda C., Touraki M. (2023). Probiotic-derived bioactive compounds in colorectal cancer treatment. Microorganisms.

[86] Xu X., Qiao Y., Peng Q., Shi B., Dia V.P. (2021). Antioxidant and immunomodulatory properties of partially purified exopolysaccharide from *Lactobacillus casei* isolated from Chinese northeast sauerkraut. Immunol. Investig..

[87] El-Dein A.N., Nour El-Deen A.M., El-Shatoury E.H., Awad G.A., Ibrahim M.K., Awad H.M., Farid M.A. (2021). Assessment of exopolysaccharides, bacteriocins and in vitro and in vivo hypocholesterolemic potential of some Egyptian *Lactobacillus* spp.. Int. J. Biol. Macromol..

[88] Fanning S., Hall L.J., Cronin M., Zomer A., MacSharry J., Goulding D., O’Connell M., Shanahan F., Nally K., Dougan G. (2012). Bifidobacterial surface-exopolysaccharide facilitates commensal-host interaction through immune modulation and pathogen protection. Proc. Natl. Acad. Sci. USA.

[89] Anukam K.C., Macklaim J.M., Gloor G.B., Reid G., Boekhorst J., Renckens B., van Hijum S.A.F.T., Siezen R.J., Planet P.J. (2013). Genome sequence of *Lactobacillus pentosus* KCA1, Vaginal isolate from a healthy premenopausal woman. PLoS ONE.

[90] Peng H.L., Shiou S.R., Chang H.Y. (1999). Characterization of mdcR, a regulatory gene of the malonate catabolic system in *Klebsiella pneumoniae*. J. Bacteriol..

[91] Schell M.A. (1993). Molecular biology of the LysR family of transcriptional regulators. Annu. Rev. Microbiol..

[92] Chohnan S., Akagi K., Takamura Y. (2003). Functions of malonate decarboxylase subunits from *Pseudomonas putida*. Biosci. Biotechnol. Biochem..

[93] Chohnan S., Takamura Y. (2004). Malonate decarboxylase in bacteria and its application for determination of intracellular acyl-CoA thioesters. Microbes Environ..

[94] Maldonado-Barragán A., Caballero-Guerrero B., Lucena-Padrós H., Ruiz-Barba J.L. (2011). Genome sequence of *Lactobacillus pentosus* IG1, a strain isolated from Spanish-style green olive fermentations. J. Bacteriol..

[95] Cronan J.E., Waldrop G.L. (2002). Multi-subunit acetyl-CoA carboxylases. Prog. Lipid Res..

[96] Bachhawat A.K., Yadav S. (2018). The glutathione cycle, glutathione metabolism beyond the γ-glutamyl cycle. IUMBM Life.

[97] Niehaus T.D., Elbadawi-Sidhu M., de Crécy-Lagard V., Fiehn O., Hanson A.D. (2017). Discovery of a widespread prokaryotic 5-oxoprolinase that was hiding in plain sight. J. Biol. Chem..

